# Lethal and sublethal influence of triflumezopyrim on life history traits and enzyme activities in the bean bug, *Riptortus pedestris* (Hemiptera: Alydidae)

**DOI:** 10.1371/journal.pone.0361771

**Published:** 2026-10-29

**Authors:** Gefan Tian, Jianglong Guo, Jingjie An, He Yuan, Zhixu Hu, Yaofa Li, Zhanlin Gao

**Affiliations:** 1 Plant Protection Institute, Hebei Academy of Agriculture and Forestry Sciences/National Collection of Plant-associated Microbes (Hebei)/IPM Innovation Center of Hebei Province/ International Science and Technology Joint Research Center on IPM of Hebei Province, Baoding, China; 2 Hebei Academy of Agriculture and Forestry Sciences, Shijiazhuang, China; Government College University Faisalabad, PAKISTAN

## Abstract

*Riptortus pedestris* (Fab.) is an important soybean pest in East Asia, where its piercing-sucking feeding behavior leads to soybean syndrome. Triflumezopyrim, a novel mesoionic insecticide, has been widely applied to control sap-sucking insects. However, its lethal and sublethal impacts on *R. pedestris* remain poorly understood. This study investigated the lethal and sublethal effects of triflumezopyrim on *R. pedestris* using bioassays, age-stage two-sex life table analyses, and enzyme activity measurements. Results demonstrated that triflumezopyrim exhibited strong toxicity (LC_50_ = 7.46 mg/L) and notable repellent activity against *R. pedestris*. Exposure of nymphs to sublethal concentrations (LC_10_ and LC_30_) significantly prolonged nymphal development and the adult preoviposition period, while reducing adult longevity, oviposition duration, and fecundity in the parental (F_0_) generation. In addition, LC_30_ exposure produced adverse transgenerational effects in the F_1_ generation, including extended adult preoviposition period, decreased male longevity, reduced oviposition duration, fecundity, net reproductive rate (*R*_*0*_), intrinsic rate of increase (*r*), and finite rate of increase (*λ*) of the population. Moreover, both LC_10_ and LC_30_ treatments led to substantial elevation in the activities of antioxidant (superoxide dismutase and peroxidase) and detoxification (multifunctional oxidase and glutathione S-transferase) enzymes. These findings indicate that triflumezopyrim exerts strong acute toxicity and pronounced sublethal inhibitory effects on *R. pedestris*, ultimately suppressing population growth potential. This study offers a scientific basis for the rational use of triflumezopyrim as well as the development of strategies for the management of this species.

## Introduction

The bean bug, *Riptortus pedestris* (Fabricius) (Hemiptera: Alydidae), is a destructive piercing-sucking pest found across China, Korea, Japan, and other regions of Asia [1–3]. This species feeds on approximately 30 host plants belonging to 13 families, including cowpea, rice, and fruit trees, with a marked preference for legumes, particularly soybean [4]. As a hemimetabolous insect, both nymphs and adults share similar feeding habits, damaging plant leaves, stems, pods, and seeds through piercing and sap extraction, which leads to substantial reductions in yield and quality [5]. Infestation typically occurs between the flowering and harvesting stages [6]. Recently, increasingly severe *R. pedestris* infestations have occurred in the Huang-Huai-Hai region, where it is recognized as a major contributor to soybean stay-green syndrome [3,7], a condition analogous to “stem green syndrome” reported in Japan and the United States [8,9]. Currently, no insecticides are specifically registered for controlling *R. pedestris* in China, and management relies largely on broad-spectrum compounds such as lambda-cyhalothrin and emamectin benzoate [10]. However, these insecticides often exhibit high toxicity toward beneficial arthropods, including parasitoids and predators [11]. Therefore, there is a pressing need to identify more selective and environmentally compatible control agents for this pest.

The recently developed mesoionic insecticide, triflumezopyrim (TFM), acts on the nicotinic acetylcholine receptor (nAChR) [12]. Unlike neonicotinoids and other receptor agonists, TFM functions as an nAChR inhibitor, competitively binding to the orthosteric site and thereby blocking neural transmission. This mode of action results in insect lethargy, paralysis, and eventual death [12]. The high efficacy of TFM against a wide range of sap-feeding pests has been proven, including leafhopper populations resistant to conventional insecticides such as imidacloprid [12,13]. In addition, it exhibits relatively low toxicity toward non-target organisms, including natural enemies such as *Cyrtorhinus lividipennis* and *Anagrus nilaparvatae* [14]. Since its registration in 2017, TFM has been widely used in rice systems for controlling planthoppers in China. However, its effectiveness against *R. pedestris* has not yet been systematically evaluated.

In field conditions, insect populations often face exposure to sublethal concentrations of insecticides resulting from photodegradation, temperature fluctuations, and rainfall [15]. Although such exposures may not cause immediate mortality, they can significantly influence insect biology, behavior, and physiology. For example, sublethal effects may alter developmental duration, reproductive output, feeding behavior, oviposition patterns, oogenesis, and sperm function, thereby either suppressing or enhancing population growth [16–18]. Consequently, evaluation of insecticide performance should extend beyond traditional toxicity metrics such as the median lethal dose (LD_50_) or lethal concentration (LC_50_) to include sublethal effects, which are highly relevant for integrated pest management (IPM) [15]. Over the past decades, sublethal effects have been extensively investigated in both pest and beneficial insects across multiple insecticide classes, including pyrethroids [19], neonicotinoids [17,20], and diamides [21]. For TFM, existing studies have primarily focused on rice planthoppers such as *Nilaparvata lugens* [22], *Laodelphax striatellus* [23], and *Sogatella furcifera* [24]. In contrast, information regarding its sublethal effects on *R. pedestris* remains limited.

Antioxidant and detoxification enzymes play essential roles in the physiological responses of insects to environmental stressors, including insecticide exposure. Antioxidant enzymes, such as superoxide dismutase (SOD), peroxidase (POD), and catalase (CAT), facilitate the removal of high levels of reactive oxygen species (ROS) generated during metabolic processes, thereby maintaining cellular redox homeostasis and protecting against injury [25]. Detoxification enzymes, including multifunctional oxidase (MFO), carboxylesterase (CarE), and glutathione S-transferase (GST), are involved in the metabolism and detoxification of xenobiotics, reducing their toxicity [26]. Exposure to sublethal concentrations of insecticides can modulate the activity of these enzyme systems, potentially contributing to resistance [27,28]. Accordingly, enzyme activity profiles are widely used as biomarkers to assess insect responses to sublethal stress.

In light of these considerations, the present study aimed to comprehensively evaluate the lethal and sublethal effects of TFM on *R. pedestris* – a pest for which such information remains lacking. Specifically, we (i) assessed the acute toxicity and repellent activity of TFM against third-instar nymphs; (ii) examined its sublethal impacts on the reproduction, development, and longevity of the parental (F_0_) generation using age-stage, two-sex life table analysis; (iii) investigated whether these effects extend to the F_1_ generation in the absence of direct exposure; and (iv) quantified the activities of key antioxidant and detoxification enzymes to reveal underlying physiological responses. These findings provide a scientific basis for the rational use of TFM and contribute to the development of effective management strategies for *R. pedestris*.

## Materials and methods

### Insects and insecticides

The population of the bean bug, *R. pedestris*, was obtained from soybean field in Shijiazhuang, Hebei Province, China (38°19′ N, 114°7′ E) in August 2019 to establish a laboratory colony. The colony was subsequently housed under controlled conditions at 26 ± 1°C and 60 ± 5% relative humidity, with 16 h light and 8 h dark. No insecticide exposure occurred during colony maintenance. Both nymphs and adults were reared on soybean seedlings supplemented with dried soybean seeds [29]. Technical-grade TFM (96% purity) was supplied by Corteva Agriscience (Shanghai, China).

### Bioassays

The toxicity of TFM to third-instar nymphs was assessed with a seedling-dip method reported previously [30]. Briefly, a stock solution of TFM (50 mg/mL) was prepared by initial dilution in acetone and subsequent addition of distilled water with 0.1% (v/v) Tween-80–20, 10, 5, 2.5, 1.25, or 0.625 mg/L TFM. Soybean seedlings bearing cotyledons were immersed in each solution for 20 s and then air-dried at room temperature. The roots of treated seedlings were wrapped in absorbent cotton and placed into plastic containers. Third-instar nymphs were then introduced and allowed to feed on the treated plants. Seedlings treated with water containing 0.1% (v/v) Tween-80 served as the control. Each concentration was replicated four times, with 20 nymphs used per replicate. Mortality was recorded after 72 h of exposure.

### Behavioral response trial

The behavioral responses of third-instar nymphs to TFM were evaluated using a Y-tube olfactometer, consisting of a main tube (25 cm) and two lateral arms (20 cm each) angled at 60°. Each arm was connected to a separate odor source chamber. A TFM-treated soybean seedling (LC_70_, LC_50_, LC_30_, LC_10_) was placed in one chamber, while the other contained a control seedling treated with 1% Tween-80 in water. The assay procedure has been described in a prior publication [30]. Briefly, individual third-instar nymphs that had been starved for 24 h were introduced at the bottom of the central arm and their behavior recorded for up to 5 min. Individuals that failed to select an arm within this period were classified as “no choice.” A valid choice was defined as movement at least two-thirds of the way into one arm with a residence time exceeding 5 s. To minimize positional bias, the two odor sources were switched after every five trials, and a new Y-tube apparatus was used after every 10 individuals. Each nymph was tested only once, and a total of 100 individuals were evaluated per treatment.

### Sublethal influence of TFM on the parental generation

Based on the bioassay results, LC_10_ and LC_30_ values at 72 h were selected to assess sublethal effects, with 0.1% Tween-80 in water representing the control. All groups included four replicates of 50 nymphs. Soybean seedlings were treated as stated above. After 72 h, nymphs that had survived were individually transferred into plastic containers and reared on untreated soybean seedlings and dried seeds. Developmental duration and survival were monitored each day until adulthood. Males and females emerging on the same day were paired at random and maintained in containers. Adult survival and reproductive output were monitored daily until all individuals had died. Each treatment included three replicates, with 20 mating pairs per replicate.

### Transgenerational influence on the F_1_ generation

Offspring derived from the F_0_ generation (LC_10_, LC_30_, and control groups) were used to evaluate transgenerational effects. The experimental design was based on a previously described approach [30]. From each group, 100 eggs that had been laid on the same day were selected at random and placed individually in plastic containers, with each egg treated as an independent replicate. After hatching, individuals were reared on insecticide-free soybean seeds and seedlings. Developmental duration, survival, and reproductive parameters were monitored each day until death, after which age-stage, two-sex life tables were constructed for the F_1_ generation.

### Enzyme activity assays

After molting, third-instar nymphs < 24 h old were exposed to LC_10_, LC_30_, or control treatments (0.1% Tween-80 solution). After 12, 24, 48, and 72 h of exposure, surviving individuals were collected for biochemical analyses. Nymphs were homogenized in 0.1 mM phosphate buffered saline (pH 7.4) at a defined ratio (weight (g) to volume (mL) = 1:9) using a mortar under ice-cold conditions. Homogenates were centrifuged (12,000 rpm, 10 min, 4°C), followed by quantification of protein levels and enzyme activities in supernatants [31]. Each treatment included three biological replicates (10 nymphs/replicate).

Protein concentrations and SOD, POD, CAT, MFO, CarE, and GST activities were determined using corresponding commercial assay kits (Total protein assay kit, A045-3; Total superoxide dismutase assay kit, A001-1–1; Peroxidase assay kit, A084-1–1; Catalase assay kit, A007-1–1; Multifunctional oxidase activity assay kit, A162-1–1; Carboxylesterase test kit, A133-1–1; Glutathione S-transferases assay kit, A004-1–1). All kits were obtained from Nanjing Jiancheng Bioengineering Institute (Nanjing, China).

### Statistical analyses

Lethal and sublethal concentrations (LC_10_, LC_30_, LC_50_, and LC_70_) for *R. pedestris* were estimated using a log-probit model [32]. Behavioral response data were analyzed using chi-square tests based on an expected 50:50 distribution. For the F_0_ generation, developmental duration, longevity, preoviposition period, oviposition period, and fecundity were assessed using one-way analysis of variance (ANOVA) with Tukey’s multiple comparison test (*P* < 0.05). Data normality and homogeneity of variance were verified using the Kolmogorov-Smirnov test and Levene’s test, respectively. These analyses were conducted using SPSS 20.0 (IBM, Armonk, NY, USA). For the F_1_ generation, life history parameters were analyzed using the TWOSEX-MSChart software [33] based on the age-stage, two-sex life table theory [34,35]. Standard errors were determined from 100,000 bootstrap resampled samples. Paired bootstrap tests were utilized for comparisons among the LC_10_, LC_30_, and control groups (*P* < 0.05). GraphPad Prism 14.0 (GraphPad Software, MA, USA) was utilized for generating illustrations.

## Results

### Toxicity of TFM to *R. pedestris* nymphs

Probit analysis revealed that 50% of the third instars of *R. pedestris* subjected to TFM would die under laboratory conditions (LC_50_) at 7.46 mg/L (95%CI: 6.03‒9.15 mg/L) (Table 1). Other proportions that will follow similar fate at 10%, 30% and 70% (corresponding to LC_10_, LC_30_, and LC_70_) are shown in Table 1.

**Table 1:** Toxicity of triflumezopyrim to 3rd instar nymphs of *R. pedestris.*

Insecticide	Slope ± SE^a^	LC_10_ mg L^-1^ (95% CI)^b^	LC_30_ mg L^-1^ (95% CI)^b^	LC_50_ mg L^-1^ (95% CI)^b^	LC_70_ mg L^-1^ (95% CI)^b^	*χ* ^*2*^	*P*
Triflumezopyrim	1.94 ± 0.23	1.63 (0.96-2.31)	4.00 (2.94-5.03)	7.46 (6.03-9.15)	13.91 (11.21-18.35)	0.52	0.92

^a^Standard error.

^b^95% confidence interval.

### Repellent effects of TFM

Treating soybean seedlings with TFM elicited a concentration-dependent behavioral response in third-instar nymphs, indicating clear repellency at higher doses. Compared with the control, nymphs showed significant avoidance of seedlings treated at LC_50_ (*χ2* = 6.37, *df* = 1, *P* = 0.012) and LC_70_ concentrations (*χ2* = 10.71, *df* = 1, *P* = 0.001). In contrast, no statistically significant behavioral differences were observed at either LC_10_ (*χ2* = 0.01, *df* = 1, *P* = 0.92) or LC_30_ (*χ2* = 2.65, *df* = 1, *P* = 0.10) concentrations (Fig 1).

**Fig 1:**
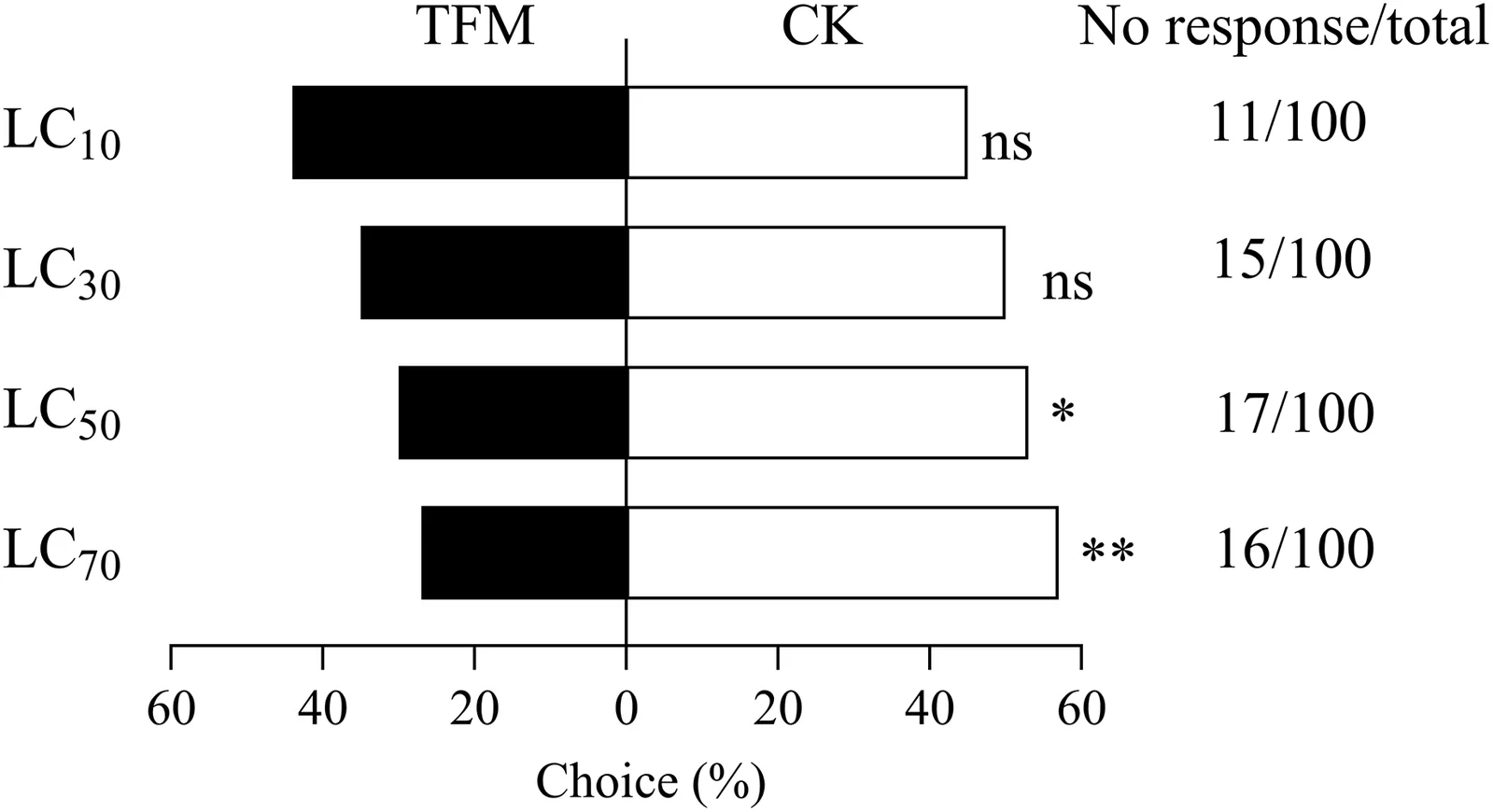
Behavioral responses of third-instar nymphs of *R. pedestris* to triflumezopyrim exposure. (*P < 0.05, **P < 0.01; chi-square test). The CK group represents seedling treated with 1% Tween-80 water solution.

### Sublethal influence of TFM on F_0_ reproduction and development

There was a significant fitness cost on development and reproductive performance on F_0_ generation following TFM exposure. Compared to the control, the durations of the third (*F*_*2, 508*_ = 38.19, *P* < 0.001), fourth (*F*_*2, 473*_ = 35.69, *P* < 0.001), and fifth (*F*_*2, 451*_ = 26.18, *P* < 0.001) instar stages were substantially extended in individuals exposed to LC_10_ and LC_30_ treatments (Table 2). Adult longevity was also markedly reduced, with significant decreases observed in both males (*F*_*2, 132*_ = 25.83, *P* < 0.001) and females (*F*_*2, 132*_ = 23.35, *P* < 0.001) compared with the control group (Table 2). In addition, the APOP differed significantly among treatments (*F*_*2, 132*_ = 9.38, *P* < 0.001). The longest APOP was recorded in the LC_30_ group (6.51 ± 0.15 days), whereas the shortest occurred in the control group (5.62 ± 0.17 days) (Table 2). Reproductive parameters were similarly affected, with both oviposition duration (*F*_*2, 132*_ = 47.16, *P* < 0.001) and fecundity (*F*_*2, 132*_ = 29.07, *P* < 0.001) significantly reduced in TFM-treated groups relative to the control (Table 2).

**Table 2:** Sublethal influence of triflumezopyrim on the development and reproduction of the F_0_ generation of *R. pedestris.*

Stages or Parameters	Control (CK)	Triflumezopyrim LC_10_	Triflumezopyrim LC_30_
3rd instar (d)	5.12 ± 0.08 c	5.78 ± 0.09 b	6.22 ± 0.09 a
4th instar (d)	4.72 ± 0.09 b	5.70 ± 0.10 a	5.68 ± 0.10 a
5th instar (d)	6.56 ± 0.08 c	7.02 ± 0.09 b	7.50 ± 0.10 a
Female longevity (d)	84.11 ± 1.84 a	72.16 ± 1.84 b	66.44 ± 1.91 b
Male longevity (d)	87.27 ± 1.55 a	79.67 ± 1.44 b	71.11 ± 1.76 c
APOP^a^ (d)	5.62 ± 0.17 b	6.40 ± 0.16 a	6.51 ± 0.15 a
Oviposition days (d)	66.31 ± 1.63 a	56.58 ± 1.71 b	43.38 ± 1.69 c
Fecundity (eggs/female)	353.51 ± 10.88 a	290.00 ± 10.40 b	239.30 ± 7.30 c

^a^Adult preoviposition period. Different letters in a given row indicate significant differences based on Tukey’s HSD test (*P* < 0.05).

### Sublethal influence of TFM on F_1_ reproduction and development

In the F_1_ generation, developmental responses varied depending on the parental exposure level. Offspring derived from the LC_30_-treated group exhibited significantly prolonged durations of the second, fourth, and fifth instars, as well as the preadult period compared with the control, whereas no corresponding differences were evident for these stages when comparing LC_10_ and control insects (Table 3). No marked treatment effects were observed in eggs (LC_10_ vs CK; LC_30_ vs CK) and first (LC_10_ vs CK; LC_30_ vs CK) and third (LC_10_ vs CK; LC_30_ vs CK) instars (Table 3). Male longevity was substantially lower in the LC_30_ group, while neither male nor female longevity differed significantly in the LC_10_ treatment relative to the controls (Table 3). Both APOP and TPOP were markedly prolonged in the LC_30_ group relative to the controls, while no such effects were detected in the LC_10_ group (Table 3). Furthermore, oviposition days and fecundity were substantially lower in the LC_30_ group, while these parameters remained statistically unchanged in the LC_10_ group (Table 3).

**Table 3:** Sublethal influence of triflumezopyrim on the development and reproduction of the F_1_ generation of *R. pedestris.*

Stages or Parameters	Control (CK)	Triflumezopyrim LC_10_	Triflumezopyrim LC_30_	*P* (LC_10_ vs CK)	*P* (LC_30_ vs CK)
Egg period (d)	8.26 ± 0.05 a	8.15 ± 0.06 a	8.30 ± 0.06 a	0.08	0.63
1st instar (d)	3.05 ± 0.05 a	2.93 ± 0.06 a	3.04 ± 0.06 a	0.15	0.95
2nd instar (d)	4.88 ± 0.11 b	4.90 ± 0.11 b	5.51 ± 0.15 a	0.92	< 0.001
3rd instar (d)	5.44 ± 0.11 a	5.42 ± 0.13 a	5.47 ± 0.16 a	0.92	0.85
4th instar (d)	4.81 ± 0.11 b	4.88 ± 0.12 b	5.71 ± 0.14 a	0.65	< 0.001
5th instar (d)	6.76 ± 0.12 b	6.83 ± 0.14 ab	7.18 ± 0.16 a	0.72	0.04
Preadult (d)	33.19 ± 0.24 b	33.15 ± 0.29 b	35.20 ± 0.37 a	0.92	< 0.001
Female longevity (d)	78.20 ± 2.11 a	75.07 ± 2.42 a	73.21 ± 2.04 a	0.33	0.09
Male longevity (d)	83.30 ± 1.72 a	82.80 ± 2.02 ab	76.41 ± 2.65 b	0.84	0.03
Total longevity (d)	113.87 ± 1.37 a	112.15 ± 1.64 a	109.98 ± 1.93 1.66 a	0.42	0.07
APOP	5.51 ± 0.15 b	5.79 ± 0.16 ab	6.07 ± 0.20 a	0.21	0.03
TPOP^a^	38.89 ± 0.40 b	39.14 ± 0.47 b	41.50 ± 0.57 a	0.68	< 0.001
Oviposition days (d)	61.05 ± 1.79 a	57.14 ± 1.91 ab	54.07 ± 1.99 b	0.14	0.001
Fecundity (eggs/female)	336.14 ± 12.37 a	328.24 ± 15.85 a	260.32 ± 16.69 b	0.70	< 0.001

^a^Total preoviposition period. Different letters in a given row indicate significant differences based on paired bootstrap tests (*P* < 0.05).

### Sublethal influence of TFM on F_1_ population parameters

The impact of TFM on population-level metrics for *R. pedestris* was further evaluated using life table parameters. Compared with the controls, the net reproductive rate (*R*_*0*_) was markedly reduced in the LC_30_ group, whereas no substantial difference was observed between the LC_10_ and control groups (Table 4). Similarly, marked differences were detected between the LC_30_ and control groups for the intrinsic rate of increase (*r*), finite rate of increase (*λ*), and mean generation time (*T*) (Table 4), whereas these parameters did not differ significantly between the LC_10_ and control treatments (Table 4).

**Table 4:** Sublethal influence of triflumezopyrim on the population parameters of the *R. pedestris* F_1_ generation.

Parameters	Control (CK)	Triflumezopyrim LC_10_	Triflumezopyrim LC_30_	*P* (LC_10_ vs CK)	*P* (LC_30_ vs CK)
Net reproductive rate (*R*_*0*_)	132.191 ± 18.017 a	104.604 ± 16.800 ab	81.899 ± 13.845 b	0.26	0.03
Intrinsic rate of increase (d^-1^) (*r*)	0.086 ± 0.003 a	0.082 ± 0.003 ab	0.074 ± 0.003 b	0.33	0.007
Finite rate of increase (d^-1^) (λ)	1.090 ± 0.003 a	1.085 ± 0.004 ab	1.077 ± 0.004 b	0.33	0.007
Mean generation time (d) (T)	56.789 ± 0.645 b	56.773 ± 0.704 b	59.539 ± 0.764 a	0.99	0.006

Different letters in a given row indicate significant differences based on paired bootstrap tests (*P* < 0.05).

The age-stage survival rate (*s*_*xj*_), representing the likelihood that an individual survives from the egg stage to a specified age (*x)* and stage (*j*), showed overlapping curves for all treatments resulting from variations in development among individuals. However, peak *s*_*xj*_ values were notably lower in both LC_10_-treated (e.g., fourth instar, 57.14%; males, 32.97%; females, 31.87%) and LC_30_-treated (e.g., fourth instar, 57.30%; males, 30.34%; females, 31.46%) groups compared with control (fourth instar, 74.16%; males, 37.08%; females, 39.33%) (Fig 2). The age-specific survival rate (*l*_*x*_), representing the likelihood of survival to age *x* irrespective of the stage of development, also declined following exposure to both LC_10_ and LC_30_ treatments (Fig 3). Fecundity-related parameters were similarly affected. The age-stage fecundity (*f*_*xj*_) and population age-specific fecundity (*m*_*x*_) correspond to mean egg production at age *x*, stage *j,* and average population fecundity from the egg stage to age *x*, respectively. Relative to the control group, the *f*_*xj*_, *m*_*x*_, and *l*_*x*_*m*_*x*_ values of the F_1_ generation derived from LC_30_-treated parents were markedly reduced (Fig 3). The reproductive value (*v*_*xj*_), a reflection of the probable contribution of age *x* and stage *j* individuals to future population growth, reached a lower peak in the LC_30_ group (73.20 eggs/female/day) than in the control group (82.66 eggs/female/day), whereas no comparable reduction was observed in the LC_10_ group (Fig 3). Life expectancy (*e*_*xj*_), representing the probable remaining lifespan of individuals at a specified age and stage, was also negatively affected by TFM exposure. Peak *e*_*xj*_ values for males (81.37 days) and females (77.64 days) in the LC_30_ group were reduced relative to those of the controls (males, 87.30 days; females, 82.57 days) (Fig 4).

**Fig 2:**
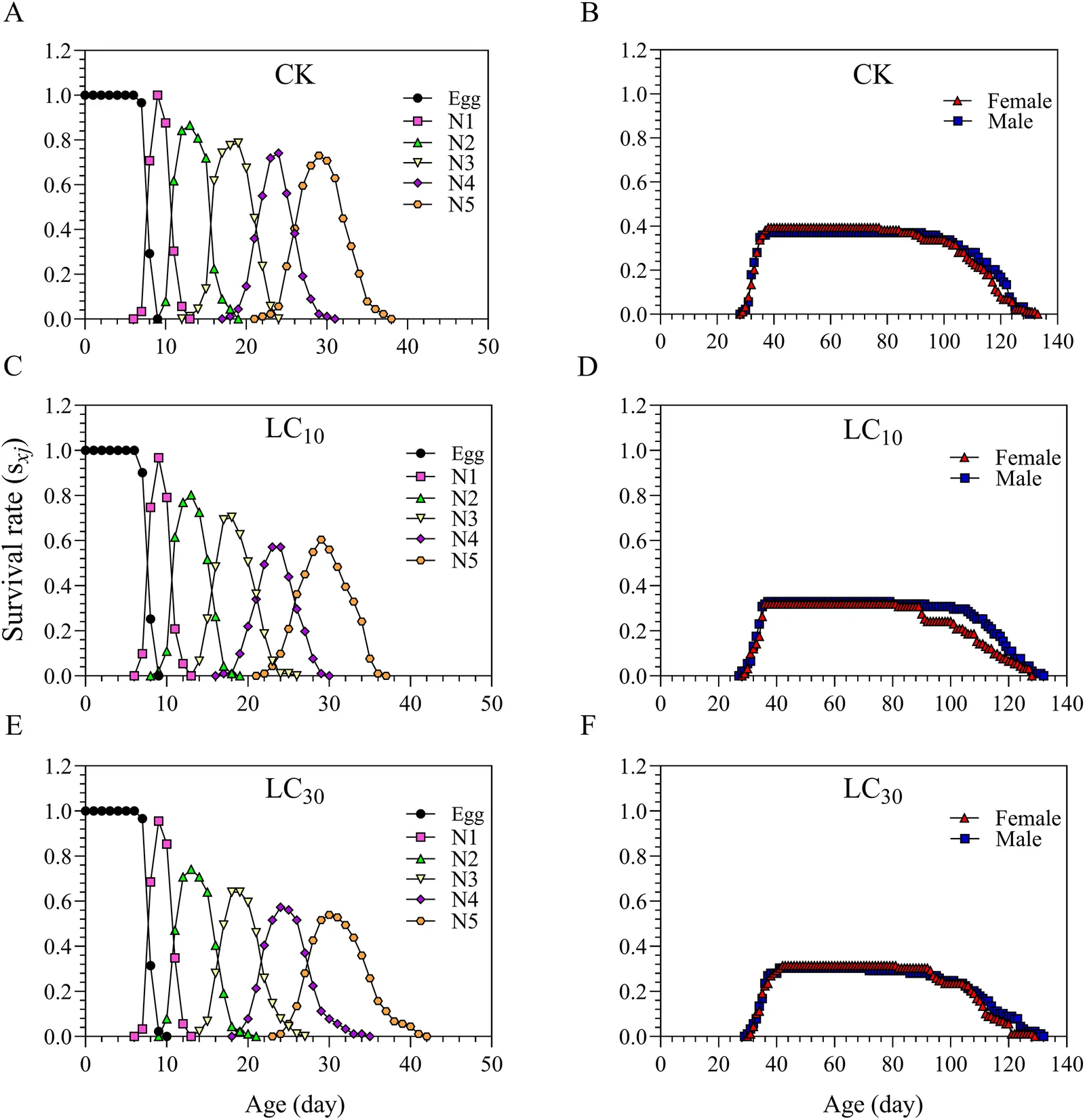
Age-stage specific survival rates (*s*_*xj*_) of F_1_ *R. pedestris* under (A, B) control conditions and following parental exposure to (C, D) LC_10_ and (E, F) LC_30_ concentrations of triflumezopyrim.

**Fig 3:**
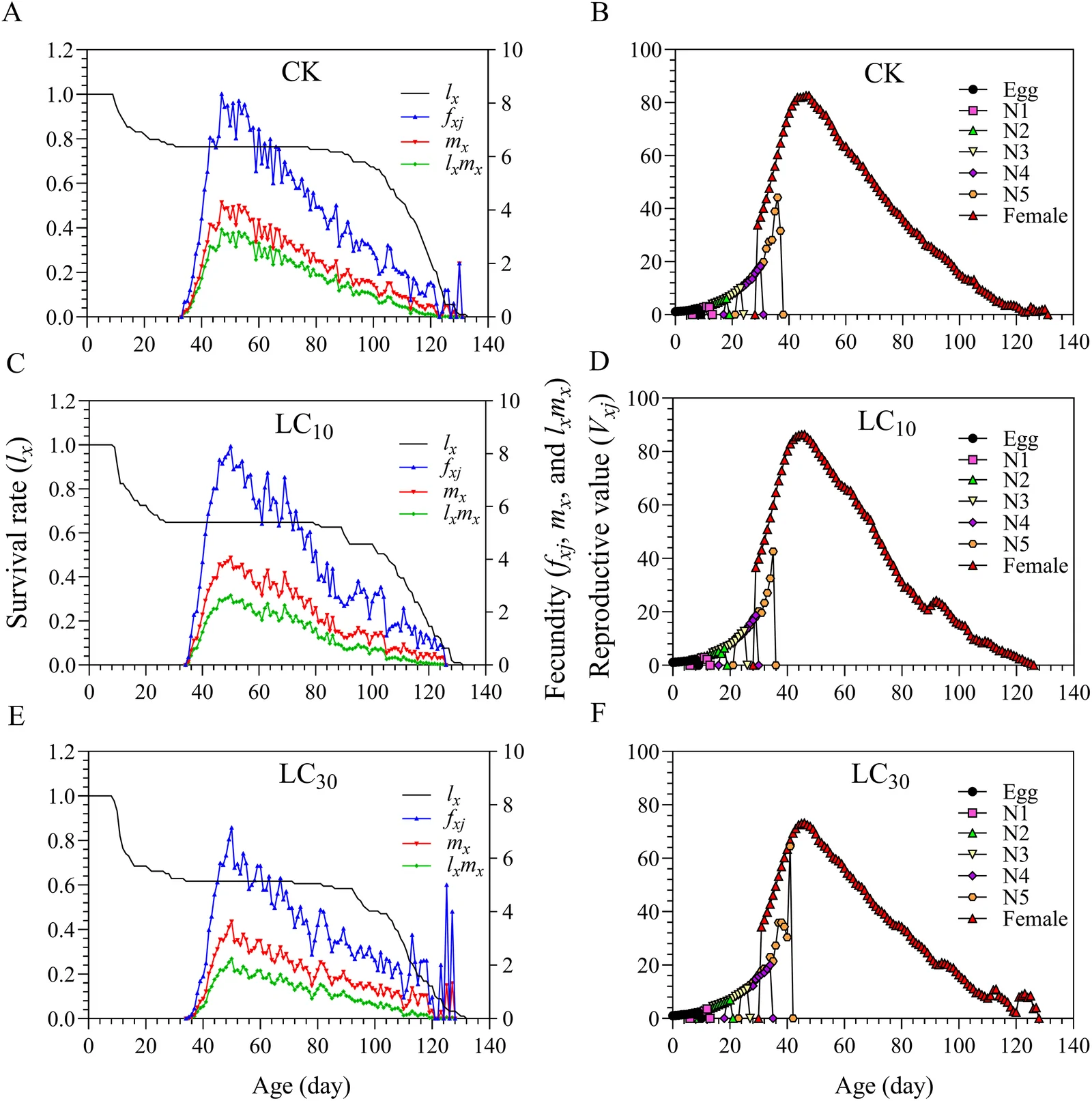
Age-specific survival (*l*_*x*_), age-stage specific fecundity (*f*_*xj*_), age-specific fecundity (*m*_*x*_), net maternity (*l*_*x*_*m*_*x*_), and reproductive value (*v*_*xj*_) of the F_1_ *R. pedestris* under (A, B) control conditions and following parental exposure to (C, D) LC_10_ and (E, F) LC_30_ concentrations of triflumezopyrim.

**Fig 4:**
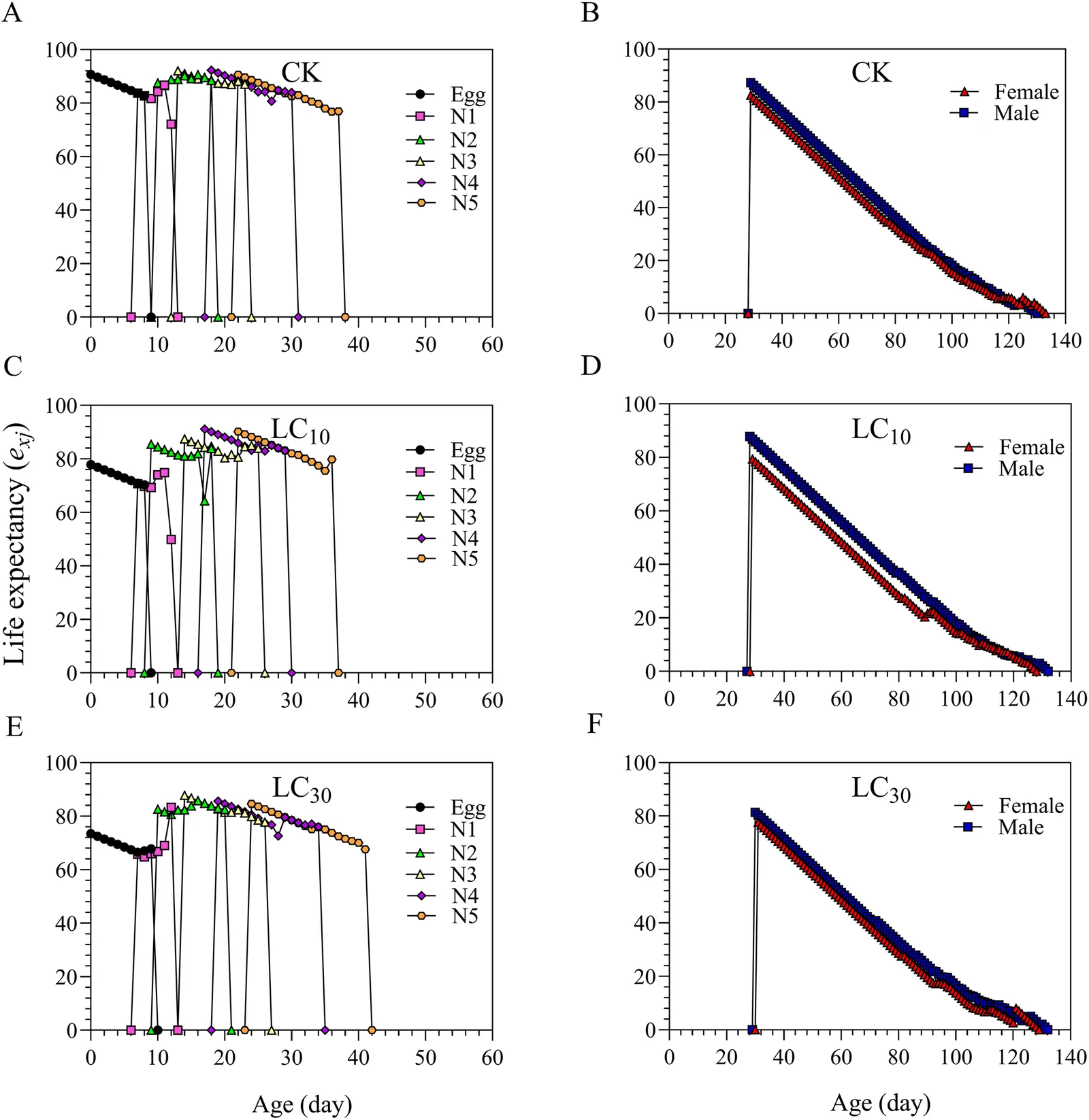
Age-stage specific life expectancy (*e*_*xj*_) of the F_1_ generation of *R. pedestris* under (A, B) control conditions and following parental exposure to (C, D) LC_10_ and (E, F) LC_30_ concentrations of triflumezopyrim.

### Sublethal influence of TFM on enzyme activity

Across the observation four periods, there were significant sublethal effects of TFM on SOD. Sublethal TFM significantly elevated SOD relative to control at 12 h (*F*_*2,* *6*_ = 88.85, *P* < 0.001), 24 h (*F*_*2, 6*_ = 277.36, *P* < 0.001), 48 h (*F*_*2, 6*_ = 314.73, *P* < 0.001), and 72 h (*F*_*2, 6*_ = 78.78, *P* < 0.001). The highest SOD activity occurred at 48 h, with 23.48% and 36.52% higher in the LC_10_ and LC_30_ groups, respectively, relative to the controls (Fig 5A). The activity of POD was also markedly elevated in both treatment groups at 24 h (*F*_*2, 6*_ = 419.68, *P* < 0.001) and 48 h (*F*_*2, 6*_ = 257.75, *P* < 0.001) compared with the control (Fig 5B). At 12 h, only the LC_10_ group showed a significant increase, whereas at 72 h, elevated POD activity was observed exclusively in the LC_30_ group (Fig 5B). The activity of CAT exhibited significant differences among treatments at 24 h (*F*_*2, 6*_ = 9.89, *P* = 0.01), 48 h (*F*_*2, 6*_ = 92.90, *P* < 0.001) and 72 h (*F*_*2, 6*_ = 54.00, *P* < 0.001), but not at 12 h (*F*_*2, 6*_ = 0.88, *P* = 0.46) (Fig 5C). Notably, opposite trends were observed between treatments such that CAT activity increased in the LC_10_ group but was suppressed in the LC_30_ group (Fig 5C).

**Fig 5:**
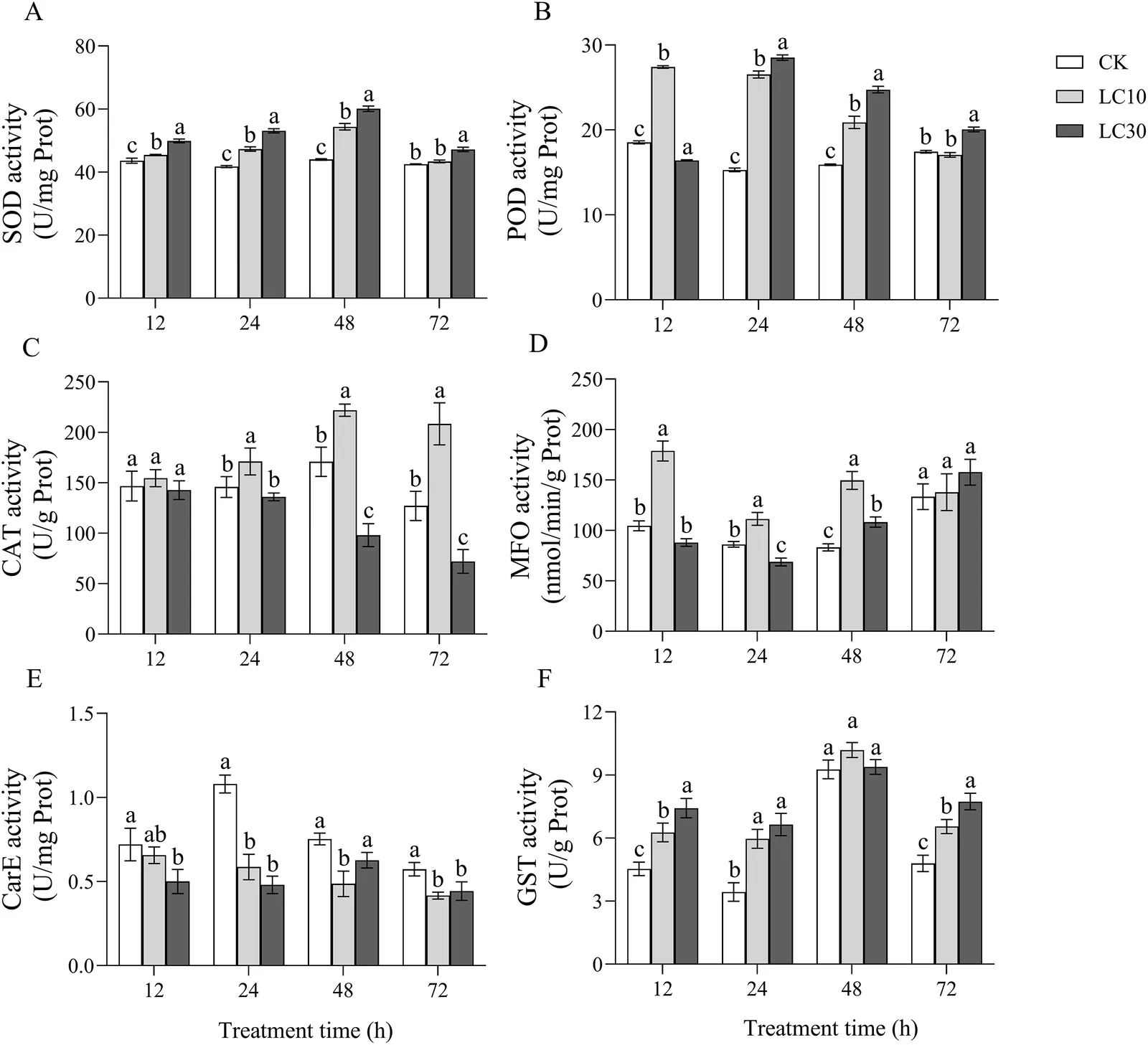
Influence of triflumezopyrim on enzyme activities in *R. pedestris* nymphs: (A) SOD, (B) POD, (C) CAT, (D) MFO, (E) CarE, and (F) GST. Different letters indicate significant differences among treatments at the same time point based on Tukey’s HSD test (*P* < 0.05).

The activity of MFO differed significantly among treatments at 12 h (*F*_*2, 6*_ = 153.89, *P* < 0.001), 24 h (*F*_*2, 6*_ = 65.23, *P* < 0.001) and 48 h (*F*_*2, 6*_ = 87.00, *P* < 0.001), but not at 72 h (*F*_*2, 6*_ = 2.29, *P* = 0.18) (Fig 5D). In the LC_10_ group, MFO activity was consistently higher than the control at 12 h, 24 h, and 48 h. In contrast, the LC_30_ group showed increased MFO activity at 48 h but a reduction relative to the control at 24 h (Fig 5D). The activity of CarE varied substantially among treatments at the different time points, including 12 h (*F*_*2, 6*_ = 6.82, *P* = 0.03), 24 h (*F*_*2, 6*_ = 82.09, *P* < 0.001), 48 h (*F*_*2, 6*_ = 17.60, *P* = 0.003), and 72 h (*F*_*2, 6*_ = 12.40, *P* = 0.007). Overall, exposure to TFM resulted in a suppression of CarE activity in *R. pedestris* (Fig 5E). The activity of GST was substantially elevated in both LC_10_ and LC_30_ at 12 h (*F*_*2, 6*_ = 37.06, *P* < 0.001), 24 h (*F*_*2, 6*_ = 38.15, *P* < 0.001), and 72 h (*F*_*2, 6*_ = 47.32, *P* < 0.001), while no significant difference was detected at 48 h (*F*_*2, 6*_ = 5.06, *P* = 0.05) (Fig 5F).

## Discussion

The present study revealed a clear progression of TFM effects on *R. pedestris*. At the toxicological level, TFM exhibited strong acute toxicity (LC_50_ = 7.46 mg/L) and concentration-dependent repellency. At the physiological level, exposure to sublethal concentrations elevated SOD, POD, MFO, and GST activities, indicating oxidative stress and detoxification activation. At the biological level, life table analyses demonstrated significant inhibition of F_0_ reproductive traits, and critically, transgenerational suppression of F_1_ population parameters (*R*_*0*_, *r*, and *λ*) following LC_30_ exposure.

Our results confirmed that TFM is highly toxic to third-instar nymphs of *R. pedestris*, with an LC_50_ of 7.46 mg/L after 72 h. This toxicity level was comparable to that of emamectin benzoate but lower than lambda-cyhalothrin, likely reflecting differences in modes of action [30]. In addition to its toxic effects, our behavioral assays revealed that TFM induced a behavioral avoidance response in *R. pedestris*, which aligns with previous observations [30]. Similar avoidance behavior has been documented in adults exposed to other insecticides, including thiamethoxam, dinotefuran, and acrinathrin, as reported by Maharjan and Jung [36], and suggests that TFM may reduce host plant colonization. From an IPM perspective, this behavioral deterrence complements the toxic effect by potentially reducing feeding damage before lethal effects manifest, although the ecological relevance of this avoidance behavior under field conditions requires further investigation.

Beyond acute toxicity, sublethal exposure to both LC_10_ and LC_30_ significantly prolonged the duration of the third, fourth, and fifth instar stages of *R. pedestris* compared with the control, with the strongest effect observed in the LC_30_ group. Adult longevity was also markedly reduced in both sexes, with females and males in the LC_30_ group living approximately 18 and 16 days shorter than their control counterparts, respectively. These findings corroborate previous reports in other insect species, where sublethal concentrations of emamectin benzoate significantly prolonged larval development in *Paederus fuscipes* [37], and thiamethoxam reduced adult longevity in *Aulacorthum solani* [38]. Similarly, our previous work demonstrated comparable developmental impairments in *R. pedestris* exposed to lambda-cyhalothrin and emamectin benzoate [30], indicating that such inhibitory effects on life history traits are a common feature across different insecticide classes. The observed developmental delay and shortened adult lifespan likely result from a combination of behavioral and physiological mechanisms. Behaviorally, TFM may suppress feeding activity, leading to reduced nutrient acquisition and slower growth [24]. Physiologically, exposure may disrupt endocrine regulation, elevate oxidative stress, and activate detoxification pathways, ultimately diverting energy away from growth and reproduction [39,40]. However, the relative contribution of these two mechanisms remains unresolved. Future studies integrating electropenetrography to quantify feeding behavior with transcriptomic analyses of stress-responsive pathways would help elucidate the causal links between TFM exposure and the observed developmental costs.

In addition to the developmental costs described above, sublethal TFM exposure caused profound disruptions to reproductive output in the parental generation. Specifically, the APOP of *R. pedestris* was significantly extended in both the LC_10_ and LC_30_ treatments, increasing from 8.2 days in control to 10.6 days and 12.1 days, respectively. More critically, fecundity declined sharply: females in the LC_30_ group produced approximately 132.4 eggs per female, which was nearly 38% lower than the 213.7 eggs observed in the control group. The oviposition period was likewise shortened, aligning with the reduced longevity reported above. These reproductive impairments are in line with a growing body of evidence on other insect pests and mites, such as reduced fecundity in *L. striatellus* exposed to TFM [23], in *A. solani* following thiamethoxam exposure [38], and in *Neoseiulus womersleyi* exposed to pyridaben [41]. Vitellogenin (*Vg*) and its receptor (*VgR*) are key molecular indicators of female reproductive capacity, and their expression levels reflect reproductive status under environmental stressors such as temperature fluctuations and insecticide exposure [19,42,43]. Based on these findings, it is plausible that TFM reduces reproductive performance in *R. pedestris* by downregulating *Vg* and *VgR* expression, as has been reported in *S. furcifera* [44], *R. pedestris* [30], and *Tuta absoluta* [45]. Testing this hypothesis will require quantifying Vg and VgR transcript levels in F_0_ females exposed to LC_10_ and LC_30_, which would establish whether the reduction in fecundity recorded here is attributable to altered vitellogenesis.

The inhibitory effects of TFM extended beyond the directly exposed generation, as evidenced by significant transgeneration suppression in the F_1_ generation. Specifically, F_1_ offspring derived from LC_30_-treated parents exhibited a significantly prolonged preadult development duration compared with the control, accompanied by a marked reduction in adult longevity and total fecundity per female. More importantly, these biological impairments translated into substantial declines in key demographic parameters, including *R*_*0*_, *r*, and *λ*. In contrast, no significant transgenerational changes were detected in the LC_10_ group, suggesting a concentration-dependent threshold for the inheritance of sublethal effects. Similar transgenerational effects have been reported in other systems, including *R. pedestris* exposed to thiamethoxam [46], *T. absoluta* treated with an LC_30_ dose of Spinosad [45], and *Myzus persicae* exposed to an LC_25_ concentration of cycloxaprid [47]. The underlying mechanisms may involve epigenetic modifications, such as DNA methylation and histone modification, as well as changes in maternal resource allocation following sublethal exposure [48]. Although transgenerational suppression was evident, its magnitude appeared reduced in the F_1_ generation, as indicated by relatively higher fecundity compared with the F_0_ generation under TFM treatment. This pattern suggests that the inhibitory effects may diminish over successive generations, a phenomenon also observed in *Aphis gossypii* [49]. Because the present study tracked only the F_1_ generation, however, whether this attenuation continues and whether population growth eventually recovers remain unknown. Tracking F_2_ and F_3_ cohorts under both continued and discontinued exposure would be required to determine how persistent the transgenerational effect is.

The biochemical assays revealed that sublethal exposure to TFM significantly perturbed the physiological homeostasis of *R. pedestris*. Compared with the control, both LC_10_ and LC_30_ treatments significantly increased SOD and POD activity in *R. pedestris*. Previous studies have demonstrated that insecticides can either stimulate or suppress antioxidant enzyme activity. For instance, chlorfenapyr significantly increased SOD activity but inhibited POD and CAT in *Paracoccus marginatus* [50]. The exposure of TFM at LC_10_ and LC_30_ enhanced SOD and POD activity in *L. striatellus* [51], while low concentrations of imidacloprid induced SOD, POD, and CAT activity in *Sclerodermus guani* [20]. In contrast, the activity of CAT exhibited a concentration-dependent response, with stimulation at LC_10_ and inhibition at LC_30_. This divergence may indicate that excessive ROS accumulation at higher concentrations impairs CAT function. Exposure to insecticides can modulate detoxification enzyme activity, either inducing or suppressing metabolic pathways [20,23], thereby impacting the resistance development over time. In the present study, MFO and GST activities were significantly elevated following exposure to both LC_10_ and LC_30_ concentrations of TFM, as previously reported [20,23]. Given the established role of cytochrome P450 enzymes in conferring TFM resistance in *L. striatellus* [52], it is likely that MFO and GST contribute substantially to TFM detoxification in *R. pedestris*. Because the insects assayed had survived exposure to LC_10_ and LC_30_, this induction also represents a metabolic cost in survivors, and its possible contribution to reduced susceptibility under repeated field application warrants monitoring.

## Conclusion

This study provides concrete evidence that TFM exhibited strong acute toxicity against third-instar *R. pedestris* nymphs together with concentration-dependent repellency. At sublethal concentrations, both LC_10_ and LC_30_ prolonged nymphal development and APOP and reduced adult longevity, oviposition duration, and fecundity in the F_0_ generation, whereas only LC_30_ suppressed F_1_ reproduction and the demographic parameters (*R*_*0*_, *r*, and *λ*). Both concentrations concurrently elevated SOD, POD, MFO, and GST activities, indicating activation of antioxidant and detoxification defenses. These results demonstrate that TFM suppresses *R. pedestris* not only through acute toxicity and behavioral deterrence but also through sublethal effects that persist into the unexposed F_1_ generation, supporting its use as a component of IPM programs for this pest. To facilitate its field adoption, future research should prioritize validating these laboratory-derived population parameters under field conditions and exploring the molecular mechanisms (e.g., vitellogenin regulation or epigenetic changes) that underpin this observed transgenerational suppression.

## References

[1] Leal WS, Higuchi H, Mizutani N, Nakamori H, Kadosawa T, Ono M. Multifunctional communication in *Riptortus clavatus* (Heteroptera: Alydidae): Conspecific nymphs and egg parasitoid *Ooencyrtus nezarae* use the same adult attractant pheromone as chemical cue. J Chem Ecol. 1995;21(7):973–85. doi: 10.1007/BF02033802 24234413

[2] Kim E, Park CG, Lim UT. Evaluation of three plant seeds as potential pre-season diets for *Riptortus pedestris*. J Asia-Pacific Entomology. 2014;17(3):521–4. doi: 10.1016/j.aspen.2014.04.013

[3] Li K, Zhang X, Guo J, Penn H, Wu T, Li L, et al. Feeding of *Riptortus pedestris* on soybean plants, the primary cause of soybean staygreen syndrome in the Huang-Huai-Hai river basin. The Crop Journal. 2019;7(3):360–7. doi: 10.1016/j.cj.2018.07.008

[4] Ahn JJ, Choi KS, Koh S. Population parameters and growth of *Riptortus pedestris* (Fabricius) (Hemiptera: Alydidae) under elevated CO_2_ concentrations. Entomol Res. 2021;51(1):12–23. doi: 10.1111/1748-5967.12479 35206688

[5] Rahman MM, Lim UT. Evaluation of mature soybean pods as a food source for two pod-sucking bugs, *Riptortus pedestris* (Hemiptera: Alydidae) and *Halyomorpha halys* (Hemiptera: Pentatomidae). PLoS One. 2017;12(4):e0176187. doi: 10.1371/journal.pone.0176187 28430798 PMC5400260

[6] Endo N, Wada T, Sasaki R. Seasonal synchrony between pheromone trap catches of the bean bug, *Riptortus pedestris* (Heteroptera: Alydidae) and the timing of invasion of soybean fields. Appl Entomol Zool. 2011;46(4):477–82. doi: 10.1007/s13355-011-0065-7

[7] Wei Z, Guo W, Jiang S, Yan D, Shi Y, Wu B, et al. Transcriptional profiling reveals a critical role of GmFT2a in soybean staygreen syndrome caused by the pest *Riptortus pedestris*. New Phytol. 2023;237(5):1876–90. doi: 10.1111/nph.18628 36404128

[8] Hill CB, Hartman GL, Esgar R, Hobbs HA. Field Evaluation of Green Stem Disorder in Soybean Cultivars. Crop Science. 2006;46(2):879–85. doi: 10.2135/cropsci2005.0207

[9] Hobbs HA, Hill CB, Grau CR, Koval NC, Wang Y, Pedersen WL, et al. Green Stem Disorder of Soybean. Plant Dis. 2006;90(4):513–8. doi: 10.1094/PD-90-0513 30786603

[10] Gao Y, Chen JH, Shi SS. Research progress on soybean stink bug (*Riptortus pedestris*). Chin J Oil Crop Sci. 2019;41(5):804–15. doi: 10.19802/j.issn.1007-9084.2019033

[11] Rimoldi F, Fogel MN, Ronco AE, Schneider MI. Comparative susceptibility of two Neotropical predators, *Eriopis connexa* and *Chrysoperla externa*, to acetamiprid and pyriproxyfen: Short and long-term effects after egg exposure. Environ Pollut. 2017;231(Pt 1):1042–50. doi: 10.1016/j.envpol.2017.08.097 28915542

[12] Cordova D, Benner EA, Schroeder ME, Holyoke CW Jr, Zhang W, Pahutski TF, et al. Mode of action of triflumezopyrim: A novel mesoionic insecticide which inhibits the nicotinic acetylcholine receptor. Insect Biochem Mol Biol. 2016;74:32–41. doi: 10.1016/j.ibmb.2016.04.008 27130855

[13] Xi C, Ahmad S, Yu J, Zhang J, Chen Y, Zhang G, et al. Seed Coating with Triflumezopyrim Induces the Rice Plant’s Defense and Inhibits the Brown Planthopper’s Feeding Behavior. Agronomy. 2022;12(5):1202. doi: 10.3390/agronomy12051202

[14] Zhu J, Li Y, Jiang H, Liu C, Lu W, Dai W, et al. Selective toxicity of the mesoionic insecticide, triflumezopyrim, to rice planthoppers and beneficial arthropods. Ecotoxicology. 2018;27(4):411–9. doi: 10.1007/s10646-018-1904-x 29404868

[15] de Franca SM, Breda MO, Barbosa DRS, Araujo AMN, Guedes CA. The sublethal effects of insecticides in insects. Biological Control of Pest and Vector Insects. InTech. 2017. doi: 10.5772/66461

[16] Ullah F, Güncan A, Abbas A, Gul H, Guedes RNC, Zhang ZJ. Sublethal effects of neonicotinoids on insect pests. Entomol Gen. 2024;44(5):1145–60. doi: 10.1127/entomologia/2024/2730

[17] Esmaeily M, Begna T, Jang H, Kwon S, Jung C. Synergistic Effects of Amitraz and Dinotefuran on Honey Bee Health: Impacts on Survival, Gene Expression, and Hypopharyngeal Gland Morphology. Int J Mol Sci. 2025;26(14):6850. doi: 10.3390/ijms26146850 40725097 PMC12296131

[18] Xian C, Xin M, Wang X, Zhu X, Wang L, Li D, et al. Sublethal emamectin benzoate suppresses multi-transgenerational reproduction and alters bacterial community composition of *Thrips tabaci*. J Pest Sci. 2026;99(3). doi: 10.1007/s10340-026-02080-8

[19] Ju D, Liu Y-X, Liu X, Dewer Y, Mota-Sanchez D, Yang X-Q. Exposure to lambda-cyhalothrin and abamectin drives sublethal and transgenerational effects on the development and reproduction of *Cydia pomonella*. Ecotoxicol Environ Saf. 2023;252:114581. doi: 10.1016/j.ecoenv.2023.114581 36731179

[20] Li S, Kong Y-J, Sun Z-X, Peng X-Y, Li J, Cao D-D, et al. Effect of sublethal treatments of imidacloprid against Aromia bungii on its parasitoid, *Sclerodermus guani*. J Econ Entomol. 2025;118(5):2385–93. doi: 10.1093/jee/toaf164 40613746

[21] Huang L, Lu M, Han G, Du Y, Wang J. Sublethal effects of chlorantraniliprole on development, reproduction and vitellogenin gene (CsVg) expression in the rice stem borer, *Chilo suppressalis*. Pest Manag Sci. 2016;72(12):2280–6. doi: 10.1002/ps.4271 26939546

[22] Li Y, Liu J, Sun W, Liu F. Effects of triflumezopyrim on the reproduction of brown planthopper, *Nilaparvata lugens*. J Asia-Pacific Entomology. 2021;24(3):850–7. doi: 10.1016/j.aspen.2021.07.012

[23] Zhang S, Wang X, Gu F, Gong C, Chen L, Zhang Y, et al. Sublethal Effects of Triflumezopyrim on Biological Traits and Detoxification Enzyme Activities in the Small Brown Planthopper *Laodelphax striatellus* (Hemiptera: Delphacidae). Front Physiol. 2020;11:261. doi: 10.3389/fphys.2020.00261 32317981 PMC7154139

[24] Zhu J, Sun WQ, Li Y, Ge LQ, Yang GQ, Xu J. Effects of a novel mesoionic insecticide, triflumezopyrim, on the feeding behavior of rice planthoppers, *Nilaparvata lugens* and *Sogatella furcifera* (Hemiptera: Delphacidae). J Integr Agr. 2020;19(10):2488–99. doi: 10.1016/S2095-3119(20)63197-5

[25] Gill SS, Tuteja N. Reactive oxygen species and antioxidant machinery in abiotic stress tolerance in crop plants. Plant Physiol Biochem. 2010;48(12):909–30. doi: 10.1016/j.plaphy.2010.08.016 20870416

[26] Gul H, Güncan A, Ullah F, Desneux N, Liu X. Intergenerational Sublethal Effects of Flonicamid on Cotton Aphid, *Aphis gossypii*: An Age-Stage, Two-Sex Life Table Study. Insects. 2024;15(7):529. doi: 10.3390/insects15070529 39057262 PMC11277007

[27] Jiang M, Qian X, Zhou Z, Liu Y, Zhang M, Yang Y. Impacts of Sublethal Doses of Spinetoram on the Biological Traits and Detoxifying Enzymes of the Tomato Leaf Miner, *Tuta absoluta* (Lepidoptera: Gelechiidae). Insects. 2024;15(12):990. doi: 10.3390/insects15120990 39769592 PMC11676950

[28] Zhang Q, Wang F, Haq IU, Li C, Gou Y, Zhang K, et al. Comparative toxicity and enzymatic detoxification responses in *Spodoptera frugiperda* (Lepidoptera: Noctuidae) to two insecticides. Ecotoxicol Environ Saf. 2024;284:116917. doi: 10.1016/j.ecoenv.2024.116917 39182280

[29] Xu H, Zhao J, Li F, Yan Q, Meng L, Li B. Chemical polymorphism regulates the attractiveness to nymphs in the bean bug *Riptortus pedestris*. J Pest Sci. 2020;94(2):463–72. doi: 10.1007/s10340-020-01268-w

[30] Guo J, An J, Chang H, Li Y, Dang Z, Wu C, et al. The Lethal and Sublethal Effects of Lambda-Cyhalothrin and Emamectin Benzoate on the Soybean Pest *Riptortus pedestris* (Fabricius). Toxics. 2023;11(12):971. doi: 10.3390/toxics11120971 38133372 PMC10747274

[31] Li W, Naeem M, Cui J, Du G, Chen H. Toxicity and Sublethal Effects of Lambda-Cyhalothrin Insecticide on Parent and Filial Generations of *Henosepilachna vigintioctomaculata* (Coleoptera: Coccinellidae). Insects. 2025;16(3):259. doi: 10.3390/insects16030259 40338237 PMC11943386

[32] Finney DJ. Probit analysis. 3rd ed. Cambridge: University of Cambridge Press. 1971.

[33] Chi H. TWOSEX-MSCHart: a computer program for the population projection based on age-stage, two-sex life table. China: Fujian Academy of Agricultural Sciences. https://www.faas.cn/cms/sitemanage/index.shtml?siteId=810640925913080000

[34] Chi H, Liu H. Two new methods for the study of insect population ecology. Bull Inst Zool Acad Sin. 1984;24(2):225–40.

[35] Chi H. Life-table analysis incorporating both sexes and variable development rates among individuals. Environ Entomol. 1988;17:26–34. doi: 10.1093/ee/17.1.26

[36] Maharjan R, Jung C. Insecticide-mediated behavioral avoidance by bean bug, *Riptortus pedestris* (Heteroptera: Alydidae). Entomol Res. 2015;45(4):184–92. doi: 10.1111/1748-5967.12113

[37] Khan MM, Nawaz M, Hua H, Cai W, Zhao J. Lethal and sublethal effects of emamectin benzoate on the rove beetle, Paederus fuscipes, a non-target predator of rice brown planthopper, *Nilaparvata lugens*. Ecotoxicol Environ Saf. 2018;165:19–24. doi: 10.1016/j.ecoenv.2018.08.047 30173022

[38] Xing T, Lei J, Han X, Wang J, Wang X, Dai C, et al. Toxicity and sublethal effects of thiamethoxam on life-history traits, feeding behavior, and detoxifying metabolism of *Aulacorthum solani*. Crop Protection. 2025;198:107387. doi: 10.1016/j.cropro.2025.107387

[39] Hasnain A, Abbas A, Song ZJ, Mariam H, Amponsah P, Chang XL, et al. Sublethal effects of insecticides on rice planthoppers: Implications for pest management and agricultural sustainability. J Appl Entomol. 2026;150(5):649–63. doi: 10.1111/jen.70063

[40] Lv N, Ma K, Li R, Liang P, Liang P, Gao X. Sublethal and lethal effects of the imidacloprid on the metabolic characteristics based on high-throughput non-targeted metabolomics in *Aphis gossypii* Glover. Ecotoxicol Environ Saf. 2021;212:111969. doi: 10.1016/j.ecoenv.2021.111969 33561773

[41] Wei J, Li C, Song C, Yang X, Jiang C, Li Q. Effects of Sublethal Concentrations of Pyridaben on Development, Reproduction, and Vg Gene Expression in *Neoseiulus womersleyi*. Insects. 2026;17(1):116. doi: 10.3390/insects17010116 41598970 PMC12841668

[42] Yao Q, Xu S, Dong Y, Que Y, Quan L, Chen B. Characterization of Vitellogenin and Vitellogenin Receptor of *Conopomorpha sinensis* Bradley and Their Responses to Sublethal Concentrations of Insecticide. Front Physiol. 2018;9:1250. doi: 10.3389/fphys.2018.01250 30279662 PMC6154279

[43] Li X-R, Li Y, Wang W, He N, Tan X-L, Yang X-Q. LC50 of lambda-cyhalothrin stimulates reproduction on the moth *Mythimna separata* (Walker). Pestic Biochem Physiol. 2019;153:47–54. doi: 10.1016/j.pestbp.2018.11.001 30744896

[44] Zhou C, Yang X-B, Yang H, Long G-Y, Jin D-C. Effects of Sublethal Concentrations of Insecticides on the Fecundity of *Sogatella furcifera* (Hemiptera: Delphacidae) via the Regulation of Vitellogenin and Its Receptor. J Insect Sci. 2020;20(5):14. doi: 10.1093/jisesa/ieaa099 33009912 PMC7583268

[45] Ullah F, Güncan A, Gul H, Hafeez M, Zhou SX, Wang YR. Spinosad-induced intergenerational sublethal effects on Tuta *absoluta*: biological traits and related genes expressions. Entomol Gen. 2024;44(2):395–404. doi: 10.1127/entomologia/2024/2452

[46] Wang Z, Wang S, Li L, Chen L, Gao Y, Yuan M, et al. The Effect of Different Thiamethoxam Concentrations on *Riptortus pedestris* Development and Fecundity. Toxics. 2024;12(7):460. doi: 10.3390/toxics12070460 39058112 PMC11280779

[47] Zhu J, Wang L, Cui Z, Huang W, Wang Q, Wang W, et al. Lethal and Sublethal Effects of the Novel cis-Nitromethylene Neonicotinoid Cycloxaprid on the Green Peach Aphid, *Myzus persicae* (Sulzer) (Hemiptera: Aphididae). Toxics. 2025;14(1):30. doi: 10.3390/toxics14010030 41600579 PMC12846306

[48] Li Z, Li ZY, Xi YQ, Su LJ, An SH, Yin XM. Sublethal and lethal concentrations of deltamethrin suppresses the population growth of *Bactrocera dorsalis*. Entomol Gen. 2025;45(1):239–51. doi: 10.1127/entomologia/2024/2852

[49] Shang J, Yao Y-S, Zhu X-Z, Wang L, Li D-Y, Zhang K-X, et al. Evaluation of sublethal and transgenerational effects of sulfoxaflor on *Aphis gossypii* via life table parameters and 16S rRNA sequencing. Pest Manag Sci. 2021;77(7):3406–18. doi: 10.1002/ps.6385 33786972

[50] Li J-Y, Chen Y-T, Wang Q-Y, Zheng L-Z, Fu J-W, Shi M-Z. Sublethal and Transgenerational Toxicities of Chlorfenapyr on Biological Traits and Enzyme Activities of *Paracoccus marginatus* (Hemiptera: Pseudococcidae). Insects. 2022;13(10):874. doi: 10.3390/insects13100874 36292822 PMC9603968

[51] Ding WJ, Du RS, Li ZG, Liu DM, Liu C, Xue YN, et al. Effects of trifluoropyrimidine on the activity of *Laodelphax striatellus* (Fallén) detoxification-metabolic enzymes and protective enzymes. J South Agric. 2023;54(12):3581–90. doi: 10.3969/j.issn.2095-1191.2023.12.012

[52] Zhang S, Gu F, Du Y, Li X, Gong C, Pu J, et al. Risk assessment and resistance inheritance of triflumezopyrim resistance in *Laodelphax striatellus*. Pest Manag Sci. 2022;78(7):2851–9. doi: 10.1002/ps.6909 35393666

